# Corporate social responsibility and workplace health promotion: A systematic review

**DOI:** 10.3389/fpsyg.2022.1011879

**Published:** 2022-10-13

**Authors:** María-Jesús Alonso-Nuez, Miguel-Ángel Cañete-Lairla, Miguel-Ángel García-Madurga, Ana-Isabel Gil-Lacruz, Marta Gil-Lacruz, Jorge Rosell-Martínez, Isabel Saz-Gil

**Affiliations:** Departamento de Dirección y Organización de Empresas, Universidad de Zaragoza, Zaragoza, Spain

**Keywords:** corporate social responsibility, CSR, workplace health promotion, WHP, systematic review

## Abstract

The complex situation that global society is facing as a result of COVID-19 has highlighted the importance of companies committing to the principles of social responsibility. Among the internal initiatives, those related to the health of workers are, obviously, highly topical. The objective of our research is to provide concise knowledge of the relationship between workplace health promotion (WHP) and corporate social responsibility (CSR) so that the relevant specialized research was gathered in a single document that lays the foundations of its applicability. A systematic review, following the PRISMA method, has been carried out. Twenty-seven articles have been selected from the main scientific databases. Their qualitative analysis concludes that CSR and WHP are linked, have beneficial reciprocal effects, need committed leadership respectful of autonomy and voluntariness, and require the establishment of specific goals within the framework of the organizations' sustainability policies. Future studies should establish the impact of the pandemic on these aspects.

## Introduction

Globalized markets, along with increasingly unstable economic environments and changes, impose on companies the need to adopt strategies that, in addition to give them a competitive advantage over other organizations, promote their sustainability (Khediri, 2021). Therefore, organizations are exposed now to external and internal challenges such as reorganizations, financial cuts, or structural changes. Leadership and management practices have a direct effect on whether an organization responds to such changes with an improvement or deterioration in organizational health (Arnetz and Blomkvist, 2007). The concern of companies for the wellbeing of workers will facilitate their adaptation to the changes and uncertainties of today's world (Di Fabio, 2016, 2017), and will in turn allow them to help their organizations in the difficult challenges they face.

To build an ethical culture usually means to balance business desires for profit with ethical responsibilities toward employees (Bulatova, 2016): healthy organizations are characterized by both good profit and health business, and the wellbeing of workers (Grawitch and Ballard, 2016). A healthy workplace maximizes the integration of worker goals for wellbeing and company objectives for profitability and productivity (Sauter et al., 1996). Unhealthy work organizations, that are not concerned with creating the conditions that lead to enhanced wellbeing, can create enormous human and financial costs (Cooper, 1994). Therefore, organizations should be diagnosed in the same way as employees in the empirical analysis of working conditions and health (Bolin and Olofsdotter, 2019).

As stated almost 20 years ago by Wilson et al. (2004), employees' perceptions of their organization affect their perception of the working environment, the way they relate to their work and see their future, and ultimately their job adaptation, health, and wellbeing. Work engagement can be improved through implementation of an adequate management of internal Corporate Social Responsibility (CSRi) by corporate leaders (López-Concepción et al., 2022). Further, employees' support ensures effective CSR programs and policies (Ramus and Steger, 2000). They show interest in the activities of their organizations that affect external stakeholders, as they develop a positive social image (Rego et al., 2010). Employees judge the social concern embedded in their organization's actions, the outcomes that result from such actions, and how individuals, both within and outside the organization, are treated interpersonally as these actions are carried out (Aguilera et al., 2007). The external prestige generated by these external CSR initiatives translates into the identification of employees with their organizations (Hameed et al., 2016).

Employees have unique characteristics as a stakeholder group, in their own right as well as having a major influence on their organization's relationships with other stakeholders. As pointed out by Simmons (2008), Human Resources Management (HRM) is both a component and a potential facilitator of Corporate Social Responsibility (CSR). Employees seek benefits from their organizations: functional, in the form of challenging, stimulating, and satisfying work; economic, in the form of competitive compensation; psychological; and ethical. The provision of these benefits from a suitable HRM is considered indicative of a socially responsible employer, offering employees CSR values similar to those of their clients (Mason and Simmons, 2014).

In our research we focus on employees, specifically on internal CSR and its link with healthy organizations focused on workplace health promotion. Scientific production on CSR has grown exponentially in recent years, as society has become more aware of its importance, and more specifically, academic literature accumulates abundant production on the relationship between CSR and workers. Aguinis and Glavas (2012) identified, among others, conceptual frameworks that describe how the psychological needs of employees drive participation in CSR; how participation in CSR is affected by needs, such as physiological support, safety, affiliation, esteem, and self-actualization; or how self-determination theory explains that decision contexts within organizations that foster competence, relationship, and employee autonomy can also drive commitment to CSR.

There have been systematic reviews carried out relating CSR and HRM (Yue, 2016; De Stefano et al., 2018; Herrera and de las Heras-Rosas, 2020; Xiao et al., 2020), CSR and organizational psychology (Glavas, 2016) or CSR and internal stakeholders' health and wellbeing (Macassa et al., 2021), but none focuses on the relationship between internal CSR and workplace health promotion as an HRM tool. Specifically, the role of workplace health promotion in the concept of corporate social responsibility was studied by Wojtaszczyk (2008), who highlighted the importance of social dialogue and worker participation. Later, in their study “CSR and the health promotion debate” on 12 articles extracted from EBSCO, Monachino and Moreira (2014) underlined that their research only furnishes a merely preliminary framework. In particular, more studies are needed to develop evaluation approaches and practical tools on how CSR in health promotion allows to align commercial and corporate needs and elucidate which factors can promote the participation of CSR in health promotion. A preliminary search of Scopus and Web of Science was conducted and no current or underway systematic reviews on this topic were identified. It seems the right moment to synthesize such experiences and highlight the opportunities that these tough times of COVID-19 can offer. The study of external activities and external performance of CSR is easier to be faced by researchers, due to the availability of information. However, it is difficult to think of any aspect of CSR different from workplace health promotion with a deeper impact on daily wellbeing of the workforce. We are sure that the recent pandemic has provided the social researcher with abundant practical information and heterogeneity among companies to witness in a near future an upswing of empirical articles on the topic that may benefit from the present systematic review.

Companies adopt CSR because it is a “win–win” strategy in terms of added wellbeing of their employees (Singhapakdi et al., 2015). The objective of this research is therefore to provide the academy, society, and its agents with a general and sufficiently concise knowledge of the relationship between workplace health promotion and CSR, so that the relevant specialized researches was gathered in a single document that, as a summary executive, lays the foundations of its applicability. The pandemic has posed challenges to organizations with regard to CSR, but it also offers opportunities to engage in new CSR initiatives and catalyze a new era of CSR development in the long term (He and Harris, 2020). This background underlines the social and scientific relevance of our work, since the review of the initiatives that relate CSR to health promotion can contribute to its dissemination, its implementation, and its improvement, with a positive impact on the sustainability of organizations.

A systematic review has been carried out to analyze the state of the art. In fact, one of the main contributions of this manuscript is to demonstrate that although there are previous papers on HR, workplace wellbeing and CSR, different issues have been addressed in isolation, lacking a discourse that unites the different dimensions as a whole. Reviewing is a search for the whole truth, rather than just one part of it (Mulrow, 1994).

The paper is structured as follows: identification of a relevant research problem (section Introduction), theoretical background on CSR and healthy organizations (section Theoretical background), description of the methodology (Section Methodology), presentation and discussion of results (section Discussion), and, finally, conclusions and implications of the findings (section Conclusions).

## Theoretical background

Organizational health is a continuous process that result from interconnections between multiple factors (Adkins et al., 2000). For years, academics have proposed conceptual models of healthy organizations (Cox and Cox, 1993; Smith et al., 1995; Danna and Griffin, 1999). More recently, Grawitch et al. (2006) identified five key healthy workplace practices (work-life balance, employee growth and development, health and safety, employee involvement, and recognition), and Keller and Price (2011) categorized nine elements that contribute to organizational health: accountability, capabilities, coordination and control, culture and climate, direction, external orientation, innovation and learning, leadership, and motivation.

Other explanatory models around healthy organizations incorporate the organization's interaction not only with employees, but with a wide variety of stakeholders (Zwetsloot and Pot, 2004). The introduction of perspectives such as corporate social responsibility has positioned the concept of healthy organizations in a more inclusive perspective, as a possible way to get the goal proposed by Argandoña (2011) of balancing shareholder value creation with stakeholder value protection. Successful, healthy, and sustainable organizations generate processes, practices, dynamics, and work environments that favor the wellbeing of all their stakeholders (Grueso-Hinestroza, 2016). It is possible to refer to healthy organizations insofar as they achieve positive impacts on employees, customers, shareholders, suppliers, business partners, and society in general (Grueso-Hinestroza and Rey-Sarmiento, 2013). Recently Müller et al. (2021) identified the difference between the consumers' perspective on occupational health issues and responsibilities on the one hand and what companies and stakeholders believe consumers think about these issues and responsibilities on the other.

The empirical definition of healthy organizations usually focuses on employees' psychosocial health, without including the factors that could cause or maintain this health (Acosta et al., 2015), as the socio-organizational context of the workplace and the quality of work life itself (Dejoy and Wilson, 2009). Even if employees tend to experience lower levels of stress and a higher level of wellbeing when working in units that have a positive organizational climate of safety, customer service, fairness, interpersonal treatment, control, support, and effectiveness (Jex et al., 2014). Continuing to develop occupational health psychology will allow meeting the challenge of maximizing both workforce and organizational health (Adkins, 1999). Particularly important in this VUCA (Volatile, Uncertain, Complex, and Ambiguous) environment is that companies give meaning to their employee's work, understood as a sense of coherence, direction, meaning and belonging to working life (Schnell et al., 2013). Following Probst (2009), by offering secure employment when possible, improving communication, increasing employee's participation in decision-making, and maintaining a strong commitment to organizational safety during times of change, organizations can positively impact the wellbeing of their workers and families, proactively contributing to the long-term financial success and wellbeing of the organization itself.

Health promotion in the workplace has been concerned with improving the health and wellbeing of workers through programs and services that seek to improve personal health behaviors and lifestyle decisions (Dejoy and Wilson, 2009). Occupational health promotion encompasses screening activities to identify potential health risks (for example, health risk assessments); lifestyle management activities to improve health and to prevent or minimize risks, like exercise programs, healthy food proposals or, more recently, Mindfulness-Based Interventions in workplace mental health promotion (Huang et al., 2015); and lifelong learning interventions in the workplace (Poscia et al., 2016). So, health promotion programs focus not only on employees' physical and mental conditions related to their professional roles but also on their total life including, among others, family, fitness, eating, drinking, smoking, sleeping habits, and other employee behaviors (Holmqvist, 2009). Most health promotion programs adopt a medical perspective that focuses on known health risk behaviors, but few programs have still incorporated growth and development activities or principles of positive psychology (Tetrick and Winslow, 2015). A recent review of reviews about the effectiveness of workplace health promotion interventions on physical and mental health outcomes carried out by Proper and van Oostrom (2019) showed evidence for the effectiveness of workplace interventions on the prevention of weight-related outcomes as well as mental health and musculoskeletal disorders.

Workplace health promotion and wellness programs vary significantly in size and composition. Employers looking for programs “that work“ are urged to consider if their organizational culture can facilitate their success (Goetzel et al., 2014). Forward-thinking organizations are focusing on improving the health of their overall workforce through integrated strategies that include promoting health in the workplace with the support of committed leadership (Childress and Lindsay, 2006), always keeping in mind employers' views on the promotion of workplace health and wellbeing (Pescud et al., 2015). Companies with the most effective workplace health promotion programs report superior financial performance (Grossmeier et al., 2016). Future research is needed on the factors that contribute to the successful implementation of these interventions (Proper and van Oostrom, 2019).

In order to unite the objectives of the different stakeholders, CSR actions must be accompanied by upgrades of employee working conditions (Harvey, 2019). Internal CSR practices are those directly related to the physical and psychological working environment of employees: their health and wellbeing, their training and participation in the business, their equality of opportunities and their work-family relationship (Low, 2016). As its inclusion in all major CSR measurement and reporting guidelines demonstrates, Occupational Safety and Health (OSH), a broad discipline that covers among other areas the promotion and maintenance of the highest degree of physical, mental, and social wellbeing of workers, forms an integral part of CSR (Sowden and Sinha, 2005). But nevertheless, even if CSR standards broadly include issues related to OSH, other areas of working conditions have more limited coverage. It is the case of psychosocial risk factors (lack of variety at work, lack of meaning or meaningless fragmented tasks, tasks below the worker's skills, role ambiguity, role conflict, or responsibility for other people). Other risk factors such as low levels of support in problem solving and professional development, poor relationships with superiors, or lack of social support, do not appear commonly in CSR standards (Jain et al., 2014).

The development of CSR requires new proposals about how managers and workers can best approach OHS (Montero et al., 2009). Integrating OHS in CSR leads to an interesting approach that shapes and solves a number of current concerns (Cioca et al., 2014). CSR opens new opportunities to manage OHS within the organizations, to experiment with positive OHS concepts, and connect to strategic long-term OHS and CSR strategies and development (Zwetsloot and Ripa, 2012). In the path from accident prevention to the promotion of health, safety, and wellbeing at work CSR has an important role to play by inspiring transformational leaderships rational but also founded on ethics (Zwetsloot et al., 2017). In this sense, the Global Reporting Initiative adopted in 2018 a new standard requiring companies to report on their initiatives to promote the health of workers and becoming the first instrument that specifies requirements for employers in promoting the health in the workplace (Olsen, 2020).

## Methodology

A systematic review is “a review of the evidence on a clearly formulated question that uses systematic and explicit methods to identify, select and critically appraise relevant primary research, and to extract and analyze data from the studies that are included in the review” (Khan et al., 2001). Carrying out systematic reviews is undoubtedly one of the main methods of synthesis of knowledge (Grant and Booth, 2009). They address questions that could not otherwise be answered by individual studies, allow the identification of research problems for future studies, and can generate or evaluate theories about how or why phenomena occur (Page et al., 2021).

This research has been carried out according to the Preferred Reporting Items for Systematic Reviews and Meta-Analysis PRISMA protocol (Moher et al., 2009), recently updated (Page et al., 2021). PRISMA was developed by a group of experts who identified the minimum criteria for systematic reviews for high-quality scientific publications. The use of the PRISMA checklist increases their transparency (Kelly et al., 2016) and facilitates the traceability of the entire process in general and the flow of information in particular. Following the recommendations of Hartling et al. (2015), and aligned with the PRISMA proposal, the research was characterized by its transparency and the clarity of its purpose. In the case of this research, a four-stage process was followed from its purpose: identification of relevant studies, selection of studies, mapping of data, and synthesis and reporting of the results.

### Identification of studies

First, the inclusion and exclusion criteria were specified and documented. Research studies published in scientific journals and books and chapters, all written in the English language, were included. Conference proceedings were excluded because they were considered to be ongoing investigations, the final results of which usually appear in articles. The official literature, status reports and opinion pieces in magazines were not considered either because our purpose was to identify and analyze proposals based on scientific studies.

### Selection of studies

An exhaustive search of the Scopus, Proquest, and Web of Science databases was then carried out in December 2021. To construct the optimal search equation, combinations of keywords, and phrases related to occupational health promotion and corporate social responsibility were tested. The search strategy was adapted for each included database. For example, the search equation finally used on Scopus, including limiters to consider the inclusion and exclusion criteria, was: ALL (”Corporate social responsibility“ AND ”Workplace health promotion“) AND [LIMIT-TO (DOCTYPE, ”ar“) OR LIMIT-TO (DOCTYPE, ”ch“) OR LIMIT-TO (DOCTYPE, ”re“) OR LIMIT-TO (DOCTYPE, ”bk“)] AND [LIMIT-TO (LANGUAGE, ”English“)]. No date range was included.

The bibliographic search yielded 179 investigations. The results were exported to EndNote and the information specialist on the research team removed the duplicates. The detection and elimination of duplicate studies involved 34 citations, keeping 145 articles.

Subsequently, following a pilot test, titles and abstracts were screened by two independent reviewers, MAGM and AIGL, for assessment against their adaptation to the objective of the study from the perspective of its scope, opting for those generalizable contributions in a broad sense. After title and abstract screening, the researchers kept 66 articles.

The full text of the selected citations was assessed in detail by the same reviewers. Following the critical appraisal, studies that did not meet a certain quality threshold were excluded. This decision was based on the originality of their contributions (Daudt et al., 2013), their adaptation to the objective of the study, and the affirmative answer to the five criteria proposed by Dixon-Woods et al. (2006): are the aims and objectives of the research clearly stated?, is the research design clearly specified and appropriate for the aims and objectives of the research?, do the researchers provide a clear account of the process by which their findings we reproduced?, do the researchers display enough data to support their interpretations and conclusions?, is the method of analysis appropriate and adequately explicated? Any disagreements that arose between the reviewers were resolved with an additional reviewer, MISG. Forty-eight articles were excluded in this full text screening and, therefore, 18 documents were accepted.

A backward and forward snowballing process on these included articles allowed the identification of 14 more references, which were subjected to screening. This process included nine more references. At last, the number of articles included in the qualitative synthesis was 27. The list of included and non-included articles is attached as Supplementary material (Appendices I, II).

**Figure 1:**
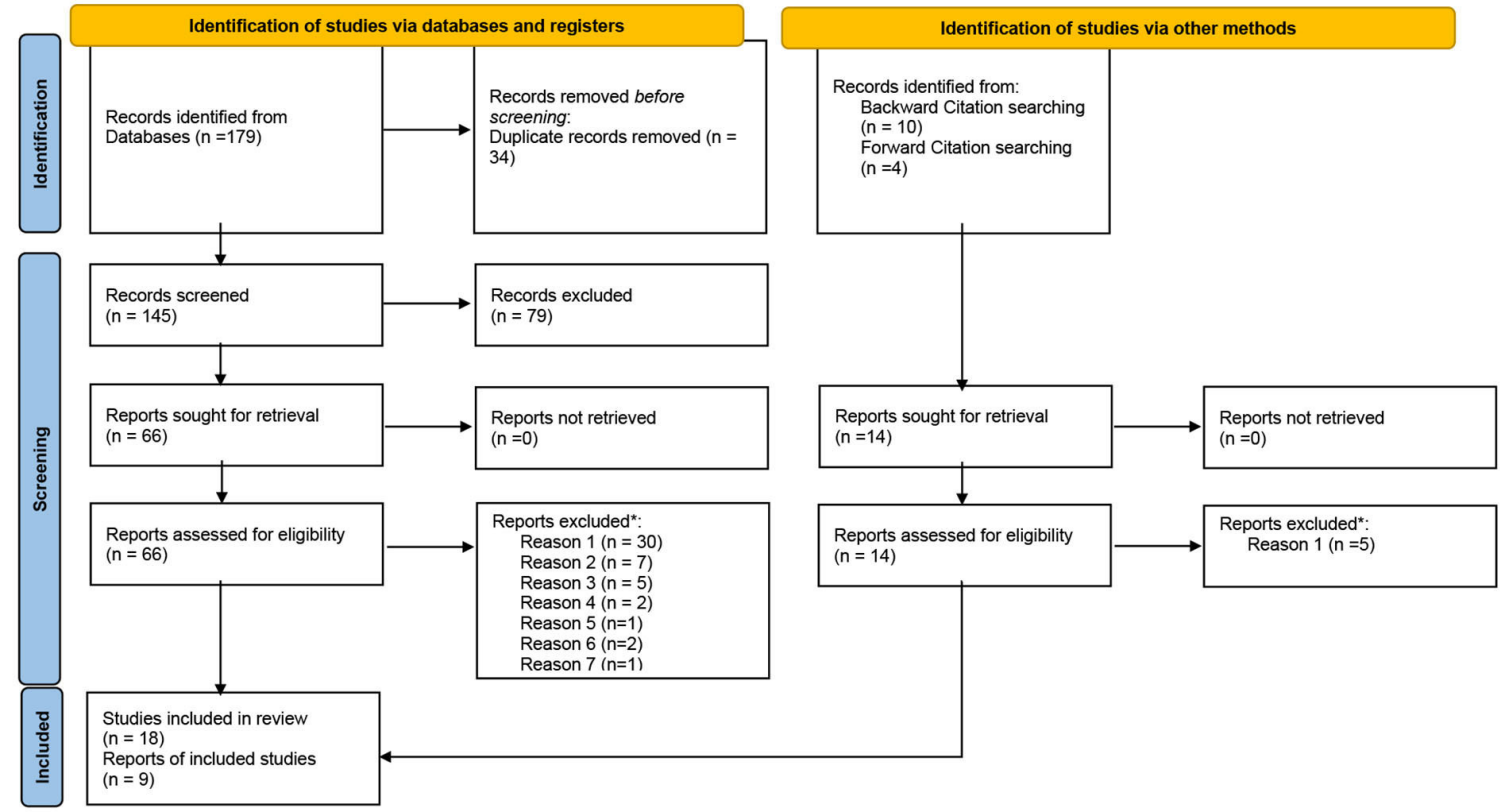
PRISMA flow diagram. *Reason 1: Non-adaptation to the objective of the study from the perspective of its scope. Reason 2: Lack of originality. Reason 3: The research design is not clearly specified and appropriate for the aims and objectives of the research. Reason 4: The researchers don't display enough data to support their interpretations and conclusions. Reason 5: The method of analysis is not appropriate and adequately explicated. Reason 6: The aims and objectives of the research are not clearly stated. Reason 7: The researchers don't provide a clear account of the process by which their findings we reproduced.

The results of the search and the study inclusion process are presented in a Preferred Reporting Items for Systematic Reviews and Meta-analyses (PRISMA) flow diagram (Figure 1) that shows the inclusion decision flowchart with the steps in the process.

### Mapping of data

All included studies underwent data extraction and synthesis. First, they were aggregated by two independent reviewers, MAGM and AIGL, in a Table 1 with all the relevant data to inform the objective and the research question. The extraction fields agreed by the research team were the following: title, first author, journal or conference, year, keywords, and main contribution. The researchers independently extracted data from the first 10 studies and pooled together to confirm that their approach to data extraction was consistent with the research question and purpose. Any disagreements that arise between the reviewers was resolved through discussion.

**Table 1:** Data extraction.

**References**	**Title**	**Journal/Book**	**Subjects**	**Country 1st** **author**	**Keywords**	**Main subject**
Auvinen et al. (2012)	Workplace health promotion and stakeholder positions: a finnish case study	Archives of Environmental and Occupational Health	Environmental Health. Occupational Health	Finland	Finland, occupational health services, stakeholders, stakeholder analysis, workplace health promotion	No stakeholders are strongly opposing the acceleration of WHP as an important part of occupational health care. There are, however, great differences in the level of interest among various stakeholders and even some resistance due to different views of OHS system development priorities and allocation of financial resources
Bamberg et al. (2019)	Enhancing organizations' social responsibility by workplace health promotion?	Social Responsibility and Sustainability	Social Responsibility. Sustainability	Germany	Workplace health promotion, corporate social responsibility, management systems, supply chain, networks	Small-sized enterprises often lack the knowledge and resources required for CSR and WHP. Therefore, they may profit crucially from support within a network
Bolis et al. (2014)	Mapping the relationships between work and sustainability and the opportunities for ergonomic action	Applied Ergonomics	Ergonomics	Brazil	Work, Sustainable development, Corporate sustainability	Ergonomics can help place the action of CSR squarely within the vision of sustainable development, at least from the standpoint of its internal social dimension
Chowdhury et al. (2021)	CSR reporting of stakeholders' health: proposal for a new perspective	Sustainability	Management, Conservation Biology	Sweden	GRI, stakeholder, health, corporate social responsibility, reporting, disclosures	Classification of core and comprehensive GRI disclosures that have direct or indirect influence on the health of external or internal stakeholders
Ferreira and de Oliveira (2014)	Does corporate social responsibility impact on employee engagement?	Journal of Workplace Learning	Human Resources	Portugal	Corporate social responsibility, employee engagement, CSR, internal CSR, external CSR, Utrecht work engagement scale	The need to enlighten the impact that socially responsible practices can have on employees' engagement
Gorgenyi-Hegyes and Fekete-Farkas (2019)	Internal CSR as a strategic management tool in reduction of labor shortages	Polish Journal of Management Studies	Management	Hungary	Labor market, sustainability, internal CSR activities, human resource management, CEE region	Potential role of internal CSR activities in reduction of labor shortages
Gorgenyi-Hegyes et al. (2021)	Workplace health promotion, employee wellbeing and loyalty during COVID-19 pandemic-large scale empirical evidence from Hungary	Economics	Development Economics and Macroeconomics	Hungary	Workplace health promotion, CSR, social sustainability, PLS-SEM, self-reliance and preservation, employee wellbeing; employee satisfaction, SDG Goal 3, COVID-19	Effects of COVID-19 pandemic on CSR activities and Health sensitivity has significantly increased due to pandemic. Important health factors have to be emphasized by not only policy decision makers but also employers
Holmqvist (2009)	Corporate social responsibility as corporate social control: The case of work-site health promotion	Scandinavian Journal of Management	Management	Sweden	preservation; employee wellbeing, employee satisfaction; SDG Goal 3, COVID-19	Health promotion may be a particularly important mechanism of corporate social control since this practice targets the very foundation of a human's “personal condition”—sickness or health; disability or fitness
Holmqvist and Maravelias (2010)	Managing healthy organizations: Worksite health promotion and the new self-management paradigm	Managing Healthy Organizations	Economics, Finance, Business, and Industry	Sweden	–	Work site health promotion may be a sign of a new or altered corporate health etic: in contrast to the old corporate health ethic, the new appears to judge the whole employee and especially what the whole employee may become
Jain et al. (2011)	Corporate social responsibility and psychosocial risk management in Europe	Journal of Business Ethics	Religion and Philosophy (General), Business (General), Ethic	UK	Psychosocial risk management, CSR, work-related stress, wellbeing, Europe	The management of psychosocial issues and risks is also about ethics and values, about doing the right things
Kuhn et al. (2020)	The ethics of workplace health promotion	Public Health Ethics	Public Health	Germany	–	Highlight the inadequacy of currently established ethical frameworks to sufficiently cover all aspects of workplace health promotion
Kuhn et al. (2021)	Interfaces of occupational health management and corporate social responsibility: a multi-center qualitative study from Germany	BMC Public Health	Health and Medicine (General)	Germany	Workplace health promotion, Corporate philosophy, ethical values, company culture, Germany	Potential, theory, and practice, for the systematic combination of OHM and CSR
Macassa et al. (2021)	Corporate social responsibility and internal stakeholders' health and wellbeing in Europe: a systematic descriptive review	Health Promotion International	Health Education	Sweden	Corporate social responsibility (CSR), internal stakeholders, health and wellbeing, Europe	The need for a consensus on measurement of internal CSR and of outcomes related to health and wellbeing, in order to enable better comparison of findings from studies across Europe
Macassa et al. (2017)	Corporate Social Responsibility and Population Health.	Health Science Journal	Health and Medicine (General)	Sweden	Corporate social responsibility, Business case, Responsible leadership, Population health	Necessity of building a platform for a joint agenda for CSR and global health promotion as part of sustainable development
Maravelias and Holmqvist (2016)	“Healthy organizations”: Developing the self-managing employee	Int. J. Human Resources Development and Management	Human Resources, Management	Sweden	workplace health promotion; WHP; health; self-management; human capital; human resource management	WHP is still a potentially precarious activity because it tends toward subordinating not only work, but also life in general to principles of management and performance
Monachino and Moreira (2014)	Corporate social responsibility and the health promotion debate: an international review on the potential role of corporations	International Journal of Healthcare Management	Medicine (General); Public aspects of medicine	Sweden	CSR, Healthcare management, Corporate communication, Health promotion, World health organization, Health partnerships	Further research was needed to develop assessment approaches and practical instruments as how a more detailed understanding of CSR involvement in health promotion may allow alignment
Moussu and Ohana (2016)	Do Leveraged Firms Underinvest in Corporate Social Responsibility? Evidence from Health and Safety Programs in U.S. Firms	Journal of Business Ethics	Religion and Philosophy (General), Business (General), Ethic	France	Debt, investment, health and safety programs, corporate social responsibility	The negative link between debt and CSR is interpreted as an efficient disciplinary effect of debt on CSR. However, the specific case of HandS programs suggests that debt discipline leads to underinvestment in activities of high importance for both firms and society
Núñez-Sánchez et al. (2021)	Corporate wellbeing programme in covid-19 times. The Mahou San Miguel case study	Sustainability	Management, Conservation Biology	Spain	corporate wellbeing, COVID-19, telewor, worker's health, physical inactivity, corporate, wellbeing programmes	Human Resources departments should adapt their workplace wellbeing programmes to the new situation
Radacsi and Hardi (2014)	Substance misuse prevention as corporate social responsibility	Substance Use and Misuse	Substance Abuse and Addiction	Hungary	Substance misuse prevention, corporate social responsibility, workplace, health promotion, East-Central Europe	Support for substance misuse prevention is an issue of both communication and a perception
Rai (2019)	Tata steel's initiatives for promotion of health and wellness through corporate social responsibility	Jharkhand Journal of Development and Management Studies	Management	India	Corporate social responsibility, sustainable business, stakeholders, economic transformation, community	Tata steel experience
Siegel et al. (2021)	Attitudes of company executives toward a comprehensive workplace health management—Results of an exploratory cross-sectional study in Germany	International Journal of Environmental Research and Public Health	Health and Medicine (General), Environmental Sciences	Germany	Workplace health management, total worker health, occupational safety and health, small and medium-sized enterprises (SME), cross-sectional survey, self-administered questionnaire, exploratory factor analysis, multiple regression analysis, Germany	Positive view on occupational safety and health according to corporate social responsibility
Sørensen and Brand (2011)	Health literacy—A strategic asset for corporate social responsibility in Europe	Journal of Health Communication International Perspectives	Health; Communication; Europe; Social Science; Public Aspects of Medicine; Social sciences (General);	The Netherlands	–	Health Literacy as an Asset for Corporate Social Responsibility
Sowden and Sinha (2005)	Promoting health and safety as a key goal of the Corporate Social Responsibility agenda	Promoting health and safety as a key goal of the Corporate Social Responsibility agenda	Health	UK	–	For the CSR movement to give OHS greater prominence it must be viewed as a material issue of reputational risk and business performance and/or an important element in the interaction of the business with employees
Tanner et al. (2019)	Workplace health promotion inspired by corporate social responsibility—Interactions within supply chains and networks	Management Revue	Management	Germany	Workplace health promotion, corporate social responsibility, supply chain, networks, collaboration	The establishing of networks is a beneficial first step and useful in spreading the idea of WHP and connecting WHP and CSR
Žižek et al. (2017)	Health-promoting leadership culture and its role in workplace health promotion	Occupational Health	Occupational Health	Slovenia	Ethics, Health, Health-promoting leadership culture, Management, Leadership, social responsibility, workplace health promotion, work environment	Leaders must be consistent and proactive about incorporating ethics into their leadership agenda, to match principles of social responsibility, including WHP
Žižek and Mulej (2016)	Creating a healthy company by occupational health promotion as a part of social responsibility	Kybernetes	Artifical Intelligence	Slovenia	Employees, health, social responsibility, Requisite holism, Work health promotion	Develop model of socially responsible occupational health and safety
Zwetsloot et al. (2013)	The core values that support health, safety, and wellbeing at work	Safety and Health at Work	Occupational Health and Safety	The Netherlands	Occupational health, organizational culture, occupational safety, social responsibility, social values	The concept of responsibility is linked with that of “prevention culture,” partly through the closely related concept of “Corporate Social Responsibility” which implies a link with business ethics

### Synthesis and reporting of the results

The publication platforms for the selected articles are mainly magazines dedicated, in this order, to Health and Medicine, Business and Management, Economics, Occupational Health, and Social Sciences. *Journal of Business Ethics* and *Sustainability* are the only journals with two articles included in the qualitative synthesis.

The keywords of the selected articles are generic for the entire corpus or specific to one or more topics. Apart from the most obvious ones, linked to the search equation, the most prominent ones are health (6), wellbeing (6), COVID-19 (3), stakeholders (3), employee satisfaction (2), Europe (2), Germany (2), human resources management (2), networks (2), occupational safety and health (2), SDG Goal 3 (2), and supply chain (2)

Regarding the geographical distribution of this literature, Sweden (7) and Germany (5) stand out, followed, with two articles, by Hungary, Slovenia, The Netherlands, and UK. It should be remembered that, according to the inclusion and exclusion criteria, literature published in languages other than English was not considered and, therefore, the analysis might not represent a global sample. Furthermore, the analysis was only performed on scientific publications, thus excluding other types of publication such as public documents and other gray literature.

## Discussion

This process involved the synthesis of findings to generate a set of statements that represent that aggregation, through assembling the findings and categorizing these findings on the basis of similarity in meaning. These categories were then subjected to a synthesis in order to produce a single comprehensive set of synthesized findings that can be used as a basis for evidence-based practice. Where textual pooling was not possible the findings were presented in narrative form. Only unequivocal and credible findings were included in the synthesis.

### Key findings

CSR and WHP are linked, and there are positive reciprocal effects between them (Bamberg et al., 2019). Work-related diseases and accidents impose high costs on the economy and have negative effects for employees and their families (Tanner et al., 2019). CSR behavior impacts on health promotion positively and so influences on occupational health and safety (Žižek and Mulej, 2016). Following Sowden and Sinha (2005), the review of the relevance of CSR for the promotion of occupational health and safety shows that, in many ways, CSR is a potentially very useful vehicle for WHP. Its focus on social outcomes, particularly the impacts on employees, should place OHS at the center of CSR. At the same time, one interesting way to improve the implementation of CSR in the organizations is to integrate CSR into specific fields of action, such as WHP (Bamberg et al., 2019), remembering that an uncontroversial issue is that companies already have a social responsibility in complying with the classic obligations of health and safety at work (Siegel et al., 2021).

Macassa et al. (2017) suggest a joint agenda for CSR and global health promotion as part of sustainable development, which would integrate, firstly, CSR and sustainability from a health perspective and, secondly, the promotion of the health of stakeholders from the perspective of sustainable development. In fact, health promotion is not only considered as a CSR activity by a growing number of corporations; also, national, and international legislators demand that companies act in a socially responsible manner through health promotion (Holmqvist, 2009).

All potential resources available should be aware of the mutual importance of CSR and WHP. However, Bamberg et al. (2019) notice that persons in charge of health promotion are rarely informed about CSR programmes. Macassa et al. (2021) highlight the need for health science researchers to be incorporated into discussions about the potential impact of internal CSR on employee health as well as on OHS outcomes beyond job satisfaction. From a health promotion perspective, organizations will be required to contribute to addressing the social determinants of health, which *per se* requires the participation of actors outside the health system (Chowdhury et al., 2021). A new type of collaboration between employees, employers, and other actors in the field is required, as WHP needs cooperation, partnerships, and alliances between both internal and external stakeholders (Auvinen et al., 2012). The WHP inspired by CSR goes beyond the limits of organizations and includes interactions with other organizations in their value chain. To spread the idea of WHP and to connect WHP and CSR, networking is a beneficial and useful first step (Tanner et al., 2019), as interactions allow sharing experiences, making decisions together or even modifying processes (Bamberg et al., 2019).

Communicating the efforts that the organizations do through standardized procedures is relevant. Chowdhury et al. (2021) synthesize the Global Reporting Initiative (GRI) contents that have a direct and indirect effect on the health of the population (internal and external stakeholders) that organizations must address when publishing their CSR reports. Open communication, transparency, and stakeholder participation link CSR and WHP (Zwetsloot et al., 2013).

### Leadership

Research such as that of Moussu and Ohana (2016) provides evidence that the decision to launch a health and safety program is taken at the CEO level. Business organizations that make the health and wellbeing of all stakeholders through CSR strategies a business case need a committed leader (Chowdhury et al., 2021). Leadership support, expressed in the participation and promotion of leaders in policies and practices that foster the development of social responsibility, is identified by Žižek et al. (2017) as an essential component of successful WHP programs. Leaders also play a relevant role in formulating goals, which is very relevant since one way to integrate CSR and WHP is to formulate clear goals (Bamberg et al., 2019), keeping in mind that the promotion of wellbeing at work is one of the main goals of companies in the context of their discourse about sustainability (Bolis et al., 2014).

Nevertheless, the findings of Jain et al. (2011) indicate that even accepting that the internal dimension of CSR has a direct relationship with occupational psychosocial risks, the “win-win” situation still seems very distant.

### Practices

Holmqvist already collected in 2008 examples of Swedish companies of work-site health promotion as a means to exercise CSR. As a good example Tetra-Pak developed activities to promote employees' health included, among others, health profiles, where employees' lifestyles were screened by professionals, and focused on so-called health-promoting leadership.

Gorgenyi-Hegyes and Fekete-Farkas (2019) group CSR initiatives within the scope of the WHP into three areas: environmental issues, like safe and secure working environment; risk factors related to nutrition, like fresh food at the workplace restaurant, cooking courses, and nutrition counseling; and risk factors related to lifestyles, like support for sport and fitness, massage, psychologist, relaxation training, screening programs, vaccinations, or regular physical check. These health preservation and health promotion initiatives affect employee loyalty through employee satisfaction and wellbeing (Gorgenyi-Hegyes et al., 2021).

In the particular case of the prevention of substance abuse in the workplace, and although CSR is valued as an adequate framework, the work of Radacsi and Hardi (2014) highlighted that it cannot be applicable in the case of employers and companies that do not even comply with their basic legal obligations regarding the safety and health of their employees.

Rai (2019) compiles the detailed description of the CSR activities carried out by the TATA firm in the field of health promotion, among which external initiatives stand out, such as the creation and operation of clinics and hospitals, health camps, family planning services and treatment and rehabilitation of people with disabilities, and interns, such as raising awareness of various health problems and generating demand for health services. More recently, Núñez-Sánchez et al. (2021) describes how Mahou San Miguel, a Spanish company whose Corporate Social Responsibility includes employees' health and wellbeing as one of its strategic lines, has adapted their corporate wellbeing programs to the new post-COVID reality.

However, as will be discussed below, within WHP settings, questions of autonomy and voluntariness are highly relevant (Kuhn et al., 2020).

### Training

CSR activities play a role for health promotion that organizations must assume, as they can focus on health education and confer understanding of the role of social determinants in health, but academics still seem to be more focused only on the involvement in health promotion of the industry sectors in which CSR strategies are considered more critical and controversial (Monachino and Moreira, 2014).

Sørensen and Brand (2011) reflect on the introduction of health literacy as an asset, as a management tool, for Corporate Social Responsibility through which the organizations can create a health-friendly environment, increase the workforce's awareness to manage their own health and make decisions in terms of promoting health and wellbeing. Values related to psychosocial issues and ethical dilemmas could, and should, also be integrated into the training plans that companies offer as part of their CSR policies (Jain et al., 2011): the more an organization actively engages in CSR practices, the more engaged their employees are (Ferreira and de Oliveira, 2014).

### Ethics

Some of the articles reviewed by Monachino and Moreira (2014) reflect a precautionary stance toward CSR, mainly warning against certain potential threats inherent in its practice, such as potential lobbying objectives and hidden agendas. As pointed out by Žižek et al. (2017), “Leaders must be consistent and proactive about incorporating ethics into their leadership agenda, to match principles of social responsibility, including WHP.” For WHP, ethical leadership is important because it is positively related to followers' ethical decision-making, prosocial behavior, and followers' satisfaction, motivation, and organizational commitment, among other factors.

Attention should also be paid to the fact that health promotion, as proposed in CSR, may be closely linked to an idea of control over the behavior and actions of workers, whose attitudes and behaviors would be shaped in accordance with company standards and value (Bolis et al., 2014); From this point of view, health promotion could be viewed as an effective means of social control “that operates both intraphysically and in terms of cultural values and norms” (Holmqvist, 2009), “closely related to critical studies of unobtrusive forms of control such as human resource management techniques and corporate culture programs” (Maravelias and Holmqvist, 2016). Participants in the study by Kuhn et al. (2021) reported difficulties in articulating ethical values relevant to both health management and CSR at the strategic level. The new corporate health ethic appears to judge the whole employee and what the whole employee may become (Holmqvist and Maravelias, 2010).

## Conclusions

The objective of our research is to provide concise knowledge of the relationship between workplace health promotion (WHP) and corporate social responsibility (CSR) so that the relevant specialized research was gathered in a single document that lays the foundations of its applicability. Among the main results obtained from the systematic review, we highlight that there is a lack of a holistic discourse on topics related to HR, labor wellbeing and CSR which support the development of evaluation approaches and practical tools on how CSR promotes health promotion. The CEO of a company that understands that the value of a company depends on its workers, will be more likely to introduce HR strategies that guarantee the access and maintenance of talented workers.

We are facing a complex and uncertain reality, in which the principles of sustainability are more valid than ever. Companies, aware of this, are redoubling their efforts within the framework of Corporate Social Responsibility. Among the internal initiatives that companies have been addressing are those related to health promotion, focused on the detection of potential health risks and the management of lifestyle for their prevention or minimization. CSR is a potentially very useful vehicle for WHP and, in parallel, WHP is a specific field of action that allows the internal deployment of social responsibility. For example, an unexpected consequence of the confinement is that for many workers the return to their workplaces has been hard. The great resignation in the USA points to the need to improve labor welfare in companies to retain workers. This paper is not devoted to the COVID 19 pandemic, but the pandemic has highlighted the importance of this research line for a wide range of stakeholders (academics, managers, workers, policy makers, and so on).

Health promotion is not only considered as a CSR activity by a growing number of companies; likewise, national, and international legislators demand that companies act in a socially responsible manner through health promotion. All potential resources available, internal, and external to the organization, must be made aware of the mutual importance of CSR and WHP. Firstly, there is a need for health science researchers to join in discussions about the potential impact of internal CSR on employee health. Secondly, a new type of collaboration between employees and employers is required. And finally, the WHP inspired by CSR includes interactions with other organizations all around the value chain and the creation of networks that allow sharing knowledge and making joint decisions from a systemic improvement approach.

The participation and promotion of leaders in policies, the formulation of objectives, and the implementation of practices that foster the development of social responsibility are essential components of successful WHP programs. To prevent these initiatives from being perceived as an attempt to control the behavior of workers and shape them to the particular values of each company, leaders must consider them in an environment of autonomy and voluntariness.

The analysis of the data obtained through a systematic review has made it possible to synthesize the key aspects of the relationship between Corporate Social Responsibility and the promotion of health at work. However, the impact of the pandemic makes us cautious about our conclusions, and future research will be necessary to determine the new relationships that will be established between companies and workers in a context that is much more concerned with aspects related to health.

## Data availability statement

The original contributions presented in the study are included in the article/Supplementary material, further inquiries can be directed to the corresponding author/s.

## Author contributions

M-JA-N, M-ÁC-L, and M-ÁG-M: conceptualization. M-JA-N, M-ÁC-L, M-ÁG-M, MG-L, JR-M, and IS-G: methodology, writing original draft, and visualization. M-ÁG-M and A-IG-L: data curation, supervision, and project administration. All authors contributed to the article and approved the submitted version.

## Funding

This research was funded by the Spanish Ministry of Economy and Competitiveness (Grant number CSO2017-82110-R) and by the Department of Science, University, and Knowledge Society of the Government of Aragón, in charge of the reference research group Wellbeing and social capital (BYCS) (ref. S16_20R, internal code 270–308).

## Conflict of interest

The authors declare that the research was conducted in the absence of any commercial or financial relationships that could be construed as a potential conflict of interest.

## Publisher's note

All claims expressed in this article are solely those of the authors and do not necessarily represent those of their affiliated organizations, or those of the publisher, the editors and the reviewers. Any product that may be evaluated in this article, or claim that may be made by its manufacturer, is not guaranteed or endorsed by the publisher.
