# Supplementary material for: Corporate social responsibility and workplace health promotion: A systematic review
Source: Front Psychol. 2022 Oct 13;13:1011879. doi: 10.3389/fpsyg.2022.1011879 (PMC9610113; doi:10.3389/fpsyg.2022.1011879)
Supplement: Supplementary file 1 [file Table_1.DOCX]

Supplementary Material

# Appendix I. Included studies

Auvinen A-M, Kohtam̈aki K, Ilvesm̈aki A. Workplace health promotion and stakeholder positions: A finnish case study. 2012;67(3):184. Available from: https://www.scopus.com/inward/record.uri?eid=2-s2.0-84867428177&doi=10.1080%2f19338244.2011.598892&partnerID=40&md5=6fd068fa52634bfc6b2d56f32dfd208d

Bamberg E, Tanner G, Baur C, Gude M. Enhancing Organizations’ Social Responsibility by Workplace Health Promotion? 2019;122. Available from: https://www.scopus.com/inward/record.uri?eid=2-s2.0-85071504999&doi=10.1007%2f978-3-030-03562-4_6&partnerID=40&md5=da9f0ee1e4242d23ed05f5c27e5e360d

Bolis I, Brunoro CM, Sznelwar LI. Mapping the relationships between work and sustainability and the opportunities for ergonomic action. 2014;45(4):1239. Available from: https://www.scopus.com/inward/record.uri?eid=2-s2.0-84899114871&doi=10.1016%2fj.apergo.2014.02.011&partnerID=40&md5=b2f81ce40458e4a8ab5dcc8e371fe572

Ehsanul Huda Chowdhury BBR and GM. CSR Reporting of Stakeholders’ Health: Proposal for a New Perspective. Sustainability. 2021;13(3):1133.

Ferreira P, de Oliveira ER. Does corporate social responsibility impact on employee engagement? 2014;26(3):247. Available from: https://www.scopus.com/inward/record.uri?eid=2-s2.0-84900850663&doi=10.1108%2fJWL-09-2013-0070&partnerID=40&md5=9ffbf2ba6613455c233937934837dff5

Gorgenyi-Hegyes E, Fekete-Farkas M. Internal csr as a strategic management tool in reduction of labour shortages. 2019;19(2):181. Available from: https://www.scopus.com/inward/record.uri?eid=2-s2.0-85069649103&doi=10.17512%2fpjms.2019.19.2.14&partnerID=40&md5=98d21d5a3d426c2e9c8c93c0993781d2

Gorgenyi-Hegyes E, Nathan RJ, Fekete-Farkas M. Workplace health promotion, employee wellbeing and loyalty during covid-19 pandemic-large scale empirical evidence from Hungary. 2021;9(2). Available from: https://www.scopus.com/inward/record.uri?eid=2-s2.0-85106501544&doi=10.3390%2feconomies9020055&partnerID=40&md5=b1099d8271c915763710bf326f76ee67

Holmqvist M, Maravelias C. Managing healthy organizations: Worksite health promotion and the new self-management paradigm. 2011;172. Available from: https://www.scopus.com/inward/record.uri?eid=2-s2.0-84917360585&doi=10.4324%2f9780203845806&partnerID=40&md5=ba36089489281e03b1b0eab1327aebcd

Holmqvist M. Corporate social responsibility as corporate social control: The case of work-site health promotion. 2009;25(1):72. Available from: https://www.scopus.com/inward/record.uri?eid=2-s2.0-60949112453&doi=10.1016%2fj.scaman.2008.08.001&partnerID=40&md5=68128a78117f2e873339cff71d7673bf

Jain A LS. Corporate social responsibility and psychosocial risk management in Europe. J Bus Ethics. 2011;101(4):619–33.

Kuhn E, Müller S, Heidbrink L, Buyx A. The ethics of workplace health promotion. 2020;13(3):246. Available from: https://www.scopus.com/inward/record.uri?eid=2-s2.0-85100317811&doi=10.1093%2fphe%2fphaa007&partnerID=40&md5=e052c4e1f8b949fa0de72300b222a744

Kuhn E, Müller S, Teusch C, Tanner G, Schümann M, Baur C, et al. Interfaces of occupational health management and corporate social responsibility: a multi-centre qualitative study from Germany. 2021;21(1). Available from: https://www.scopus.com/inward/record.uri?eid=2-s2.0-85107135998&doi=10.1186%2fs12889-021-11016-z&partnerID=40&md5=6bc764ac1af19ad335b206447a5f6aad

Macassa G MC. Corporate social responsibility and internal stakeholders’ health and well-being in Europe: a systematic descriptive review. Health Promot Int. 2020;

Macassa G. Corporate Social Responsibility and Population Health. Health Science Journal. 2017;11:528.

Maravelias C, Holmqvist M. “Healthy organisations”: Developing the self-managing employee. 2016;16(1–2):99. Available from: https://www.scopus.com/inward/record.uri?eid=2-s2.0-84962262164&doi=10.1504%2fIJHRDM.2016.075374&partnerID=40&md5=6a5479620353a4080d57f78befcc9344

Monachino MS MP. Corporate social responsibility and the health promotion debate: an international review on the potential role of corporations. International Journal of Healthcare Management. 2014;7(1):53–9.

Moussu C, Ohana S. Do Leveraged Firms Underinvest in Corporate Social Responsibility? Evidence from Health and Safety Programs in U.S. Firms. 2016;135(4):729. Available from: https://www.scopus.com/inward/record.uri?eid=2-s2.0-84917692865&doi=10.1007%2fs10551-014-2493-0&partnerID=40&md5=5b87a1a9740428ee16aff8213c3ec4c9

Núñez-Sánchez JM, Gómez-Chacón R, Jambrino-Maldonado C, García-Fernández J. Corporate well-being programme in covid-19 times. The Mahou San Miguel case study. 2021;13(11). Available from: https://www.scopus.com/inward/record.uri?eid=2-s2.0-85107879019&doi=10.3390%2fsu13116189&partnerID=40&md5=749073f040820688d5d3b20cda975d26

Radacsi G, Hardi P. Substance misuse prevention as corporate social responsibility. 2014;49(4):363. Available from: https://www.scopus.com/inward/record.uri?eid=2-s2.0-84894214738&doi=10.3109%2f10826084.2013.841242&partnerID=40&md5=e536535da38eee4284c8d12dfc3228c2

Rai K. TATA STEEL’S INITIATIVES FOR PROMOTION OF HEALTH AND WELLNESS THROUGH CORPORATE SOCIAL RESPONSIBILITY. Journal of Development and Management Studies. 2019;17(4):355–8374.

Sarotar Zizek S, Mulej M. Creating a healthy company by occupational health promotion as a part of social responsibility. Kybernetes [Internet]. 2016;45(2):243. Available from: https://www.proquest.com/scholarly-journals/creating-healthy-company-occupational-health/docview/1758123974/se-2?accountid=14795

Siegel A, Hoge AC, Ehmann AT, Martus P, Rieger MA. Attitudes of Company Executives toward a Comprehensive Workplace Health Management—Results of an Exploratory Cross-Sectional Study in Germany. International Journal of Environmental Research and Public Health [Internet]. 2021;18(21). Available from: https://www.proquest.com/scholarly-journals/attitudes-company-executives-toward-comprehensive/docview/2596019595/se-2

Sowden P SS. Promoting health and safety as a key goal of the Corporate Social Responsibility agenda. HSE Books; 2005.

Sørensen K. Health Literacy—A Strategic Asset for Corporate Social Responsibility in Europe. Journal of Health Communication International Perspectives. 2011;16(sup 3):322–7.

Tanner G, Bamberg E, Baur C, Schümann M. Workplace health promotion inspired by corporate social responsibility - Interactions within supply chains and networks. 2019;30(2–3):231. Available from: https://www.scopus.com/inward/record.uri?eid=2-s2.0-85075791224&doi=10.5771%2f0935-9915-2019-2%2f3-213&partnerID=40&md5=634882613fd588e4887653a6e4fbd38c

Zwetsloot GIJM, Scheppingen ARV, Bos EH, Dijkman A, Starren A. The core values that support health, safety, and well-being at work. 2013;4(4):196. Available from: https://www.scopus.com/inward/record.uri?eid=2-s2.0-84891372903&doi=10.1016%2fj.shaw.2013.10.001&partnerID=40&md5=4fea9d72ed3466e73c0592da74efcc18

ŽiŽek S. Health-promoting leadership culture and its role in workplace health promotion. Occup Health. 2017;1:50–99.
