# Supplementary material for: Corporate social responsibility and workplace health promotion: A systematic review
Source: Front Psychol. 2022 Oct 13;13:1011879. doi: 10.3389/fpsyg.2022.1011879 (PMC9610113; doi:10.3389/fpsyg.2022.1011879)
Supplement: Supplementary file 2 [file Table_2.DOCX]

Supplementary Material

# Appendix II. Excluded studies after full screening

Bauer GF, Jenny GJ. Moving Towards Positive Organizational Health: Challenges and a Proposal for a Research Model of Organizational Health Development. 2012;2:145. Available from: https://www.scopus.com/inward/record.uri?eid=2-s2.0-84874176794&doi=10.1002%2f9781119942849.ch8&partnerID=40&md5=b7a35a300786a0a3ba792cc6c02014ef

Reason for exclusion: Non adaptation to the objective of the study from the perspective of its scope

Bauer GF, Jenny GJ. The application of salutogenesis to organisations. 2016;224. Available from: https://www.scopus.com/inward/record.uri?eid=2-s2.0-85006741586&doi=10.1007%2f978-3-319-04600-6_21&partnerID=40&md5=270960ed7fad26ee912a15fdbaa0cd08

Reason for exclusion: Non adaptation to the objective of the study from the perspective of its scope

Bjerke R, Elvekrok I. Sponsorship-based health care programs and their impact on employees’ motivation for physical activity. 2021;21(2):217. Available from: https://www.scopus.com/inward/record.uri?eid=2-s2.0-85081330253&doi=10.1080%2f16184742.2020.1735471&partnerID=40&md5=4cb36acc9700c78462cf8c994957fb17

Reason for exclusion: Non adaptation to the objective of the study from the perspective of its scope

Brandenburg U. Volkswagen: A comprehensive approach to health promotion in the workplace. 2012;224. Available from: https://www.scopus.com/inward/record.uri?eid=2-s2.0-84949803211&doi=10.4135%2f9781446288962.n16&partnerID=40&md5=dba0106e892c1a3d295908dce61f3456

Reason for exclusion: Lack of originality

Brassart Olsen C. Towards Corporate Health Responsibility? An Analysis of Workplace Health Promotion Through the Prism of CSR and Transnational New Governance. 2020;36(1):54. Available from: https://www.scopus.com/inward/record.uri?eid=2-s2.0-85100655454&partnerID=40&md5=7e07ba5207d5bdfbe22b726669858ba3

Reason for exclusion: The aims and objectives of the research are not clearly stated

Das M, Rangarajan K, Dutta G. Corporate sustainability in small and medium-sized enterprises: a literature analysis and road ahead. 2020;12(2):300. Available from: https://www.scopus.com/inward/record.uri?eid=2-s2.0-85074339626&doi=10.1108%2fJIBR-09-2017-0166&partnerID=40&md5=5c6f89514e2dff12d2b7fb91055e746a

Reason for exclusion: The research design is not clearly specified and appropriate for the aims and objectives of the research

Day A. Small and medium sized enterprises as healthy workplaces. 2011;188. Available from: https://www.scopus.com/inward/record.uri?eid=2-s2.0-84881767229&partnerID=40&md5=5992b86bcf6bf6062fcadce56e62d268

Reason for exclusion: Lack of originality

DeJoy DM, Dyal M-A, Smith TD. Workplace health promotion: Ethical tight rope. 2018;374. Available from: https://www.scopus.com/inward/record.uri?eid=2-s2.0-85045595413&doi=10.4324%2f9781315194868&partnerID=40&md5=99a9f326f9d97ea6c3c06dd70c2b7445

Reason for exclusion: Non adaptation to the objective of the study from the perspective of its scope

Dezmar-Krainz K. Enhancing wellbeing of employees through corporate social responsibility context. Megatrend Revija. 2015;12(2):137–53.

Reason for exclusion: Lack of originality

Feißel A, Peter R, Swart E, March S. Developing an extended model of the relation between work motivation and health as affected by the work ability as part of a corporate age management approach. 2018;15(4). Available from: https://www.scopus.com/inward/record.uri?eid=2-s2.0-85045742724&doi=10.3390%2fijerph15040779&partnerID=40&md5=550b37f533f26db1a44f87b63b5fa0e8

Reason for exclusion: The researchers don´t provide a clear account of the process by which their findings we reproduced

Gabathuler M, Kirschner M. A comparison of workplace-related labels in Switzerland. 2019;12(6):423. Available from: https://www.scopus.com/inward/record.uri?eid=2-s2.0-85075735394&doi=10.1108%2fIJWHM-03-2019-0037&partnerID=40&md5=ea6b7f86dd2342e4e26a07061ee9a1cc

Reason for exclusion: Non adaptation to the objective of the study from the perspective of its scope

Garg N, Murphy WM, Singh P. Reverse mentoring and job crafting as resources for health: a work engagement mediation model. 2021; Available from: https://www.scopus.com/inward/record.uri?eid=2-s2.0-85114500060&doi=10.1108%2fJOEPP-12-2020-0245&partnerID=40&md5=faba6dfae853e319bb48bab4ae222912

Reason for exclusion: Non adaptation to the objective of the study from the perspective of its scope

Giacomini D, Martini M, Sancino A, Zola P, Cavenago D. Corporate social responsibility actions and organizational legitimacy at the peak of COVID-19: a sentiment analysis. 2021;21(6):1058. Available from: https://www.scopus.com/inward/record.uri?eid=2-s2.0-85109383303&doi=10.1108%2fCG-09-2020-0389&partnerID=40&md5=78642a0dff259fc1388a31e34bd7b459

Reason for exclusion: Lack of originality

Goetzel RZ, Fabius R, Roemer EC, Kent KB, Berko J, Head MA, et al. The Stock Performance of American Companies Investing in a Culture of Health. 2019;33(3):447. Available from: https://www.scopus.com/inward/record.uri?eid=2-s2.0-85061098330&doi=10.1177%2f0890117118824818&partnerID=40&md5=82bbe1a6cdcd55a9512722729e5ff42a

Reason for exclusion: Non adaptation to the objective of the study from the perspective of its scope

Gubler T, Larkin I, Pierce L. Doing well by making well: The impact of corporate wellness programs on employee productivity. 2018;64(11):4987. Available from: https://www.scopus.com/inward/record.uri?eid=2-s2.0-85052815935&doi=10.1287%2fmnsc.2017.2883&partnerID=40&md5=ad61766da74c6e504004975d6326782c

Reason for exclusion: The researchers don´t display enough data to support their interpretations and conclusions

Hall J, Kay T, McConnell AK, Mansfield L. Implementation of sit-stand desks as a workplace health initiative: stakeholder views. 2019;12(5):386. Available from: https://www.scopus.com/inward/record.uri?eid=2-s2.0-85071613969&doi=10.1108%2fIJWHM-02-2019-0026&partnerID=40&md5=9e728640bf79c5599937fa17ca9c09c2

Reason for exclusion: Lack of originality

Hariramani PR. THE MEDIATING EFFECT OF EMPLOYEE MOTIVATION ON THE RELATIONSHIP AND IMPACT OF CORPORATE SOCIAL RESPONSIBILITY TO EMPLOYEE RELATIONS. 2021;24(Special Issue 1):21. Available from: https://www.scopus.com/inward/record.uri?eid=2-s2.0-85118432942&partnerID=40&md5=75a742170ff1ac546f10ac89aff9e753

Reason for exclusion: Non adaptation to the objective of the study from the perspective of its scope

Harvey G. Corporate wellness: what, why not and whither? 2019;41(4):648. Available from: https://www.scopus.com/inward/record.uri?eid=2-s2.0-85067198862&doi=10.1108%2fER-06-2018-0151&partnerID=40&md5=2ad96b2a0a1b81160792445e0c10dbd8

Reason for exclusion: Non adaptation to the objective of the study from the perspective of its scope

Hendriksen IJM, Snoijer M, De Kok BPH, Van Vilsteren J, Hofstetter H. Effectiveness of a Multilevel Workplace Health Promotion Program on Vitality, Health, and Work-Related Outcomes. 2016;58(6):583. Available from: https://www.scopus.com/inward/record.uri?eid=2-s2.0-84992307628&doi=10.1097%2fJOM.0000000000000747&partnerID=40&md5=35f9696b9dccfb167ed9fb9ec42ab91d

Reason for exclusion: Non adaptation to the objective of the study from the perspective of its scope

Henke RM, Head MA, Kent KB, Goetzel RZ, Roemer EC, Mccleary K. Improvements in an Organization’s Culture of Health Reduces Workers’ Health Risk Profile and Health Care Utilization. 2019;61(2):101. Available from: https://www.scopus.com/inward/record.uri?eid=2-s2.0-85061099390&doi=10.1097%2fJOM.0000000000001479&partnerID=40&md5=9b03c52fcf3d7cc140c95ba00647446e

Reason for exclusion: Non adaptation to the objective of the study from the perspective of its scope

Herrera J, de las Heras-Rosas C. Corporate social responsibility and human resource management: Towards sustainable business organizations. 2020;12(3). Available from: https://www.scopus.com/inward/record.uri?eid=2-s2.0-85081198276&doi=10.3390%2fsu12030841&partnerID=40&md5=6add8e875fb7b574dd07588f4246cdf2

Reason for exclusion: The method of analysis is not appropriate and adequately explicated

Iavicoli S, Valenti A, Gagliardi D, Rantanen J. Ethics and Occupational Health in the Contemporary World of Work. International Journal of Environmental Research and Public Health [Internet]. 2018 Aug;15(8). Available from: https://www.proquest.com/scholarly-journals/ethics-occupational-health-contemporary-world/docview/2108433300/se-2

Reason for exclusion: Non adaptation to the objective of the study from the perspective of its scope

Jarden A, Jarden R. Positive Psychological Assessment for the Workplace. 2016;437. Available from: https://www.scopus.com/inward/record.uri?eid=2-s2.0-85019935870&doi=10.1002%2f9781118977620.ch22&partnerID=40&md5=f380e243c710f9fda0c153a89b307d45

Reason for exclusion: Non adaptation to the objective of the study from the perspective of its scope

Kawashita F TY. Occupational safety and health aspects of corporate social responsibility (CSR) in Japanese companies listed on the Tokyo stock exchange (TSE) first section. J Occup Health. 2005;47(6):533–9.

Reason for exclusion: The research design is not clearly specified and appropriate for the aims and objectives of the research

Kent KB, Goetzel RZ, Roemer EC, McCleary K, Henke RM, Head MA, et al. Developing two culture of health measurement tools examining employers’ efforts to influence population health inside and outside company walls. 2018;60(12):1097. Available from: https://www.scopus.com/inward/record.uri?eid=2-s2.0-85058570771&doi=10.1097%2fJOM.0000000000001438&partnerID=40&md5=dd4e2cc550d797864f19a52d414b4c57

Reason for exclusion: The researchers don´t display enough data to support their interpretations and conclusions

Kim Y, Park J, Park M. Creating a Culture of Prevention in Occupational Safety and Health Practice. 2016;7(2):96. Available from: https://www.scopus.com/inward/record.uri?eid=2-s2.0-84962082139&doi=10.1016%2fj.shaw.2016.02.002&partnerID=40&md5=310e483161e16e209cbd4df7d497bb63

Reason for exclusion: Lack of originality

Kukovec D, Milfelner B, Mulej M, Šarotar-žižek S. Model of socially responsible transfer of parent organization culture to the subsidiary organization in a foreign cultural environment concerning internal communication, stress and work satisfaction. 2021;13(14). Available from: https://www.scopus.com/inward/record.uri?eid=2-s2.0-85111142625&doi=10.3390%2fsu13147927&partnerID=40&md5=eba949d1f2cbabe061e941c55391dbe9

Reason for exclusion: Non adaptation to the objective of the study from the perspective of its scope

Leka S, Jain A. Policy approaches to occupational and organizational health. 2014;9789400756403:249. Available from: https://www.scopus.com/inward/record.uri?eid=2-s2.0-84932083539&doi=10.1007%2f978-94-007-5640-3_14&partnerID=40&md5=5cdfb267fb62a29e60f06c149ea788f1

Reason for exclusion: Non adaptation to the objective of the study from the perspective of its scope

Lowe G. Creating healthy organizations: How vibrant workplaces inspire employees to achieve sustainable success. 2011;258. Available from: https://www.scopus.com/inward/record.uri?eid=2-s2.0-84966762523&partnerID=40&md5=243cc485cf3e814b90e380f7848674e8

Reason for exclusion: The aims and objectives of the research are not clearly stated

Madsen CU, Hasle P. Commitment or compliance? Institutional logics of work environment management. 2017;7:38. Available from: https://www.scopus.com/inward/record.uri?eid=2-s2.0-85027714135&doi=10.18291%2fnjwls.v7iS2.96688&partnerID=40&md5=1370d96382f770cb8de5c76b196dfaa0

Reason for exclusion: Non adaptation to the objective of the study from the perspective of its scope

Miragaia DAM, Aleixo JMS. Organisational productivity: Perceptions about the influence of workplace physical activity programs on performance, wellness and worker satisfaction. 2021;15(2–3):414. Available from: https://www.scopus.com/inward/record.uri?eid=2-s2.0-85101797294&doi=10.1504%2fEJIM.2021.113266&partnerID=40&md5=5dedf9973577ff9d0bdb132dedfcc4af

Reason for exclusion: Non adaptation to the objective of the study from the perspective of its scope

Nagata T NA. . Occupational safety and health aspects of corporate social responsibility reporting in Japan from 2004 to 2012. BMC Public Health. 2017;17(1):381.

Reason for exclusion: The research design is not clearly specified and appropriate for the aims and objectives of the research

Nöhammer E, Drexel M, Stummer H. Co-Creating the good job or the extra mile: Does co-creational implementation of whp improve working conditions? 2020;31(2):258. Available from: https://www.scopus.com/inward/record.uri?eid=2-s2.0-85087625427&doi=10.5771%2f0935-9915-2020-2-232&partnerID=40&md5=b5d99059327347d40de220035767d227

Reason for exclusion: Non adaptation to the objective of the study from the perspective of its scope

Nöhammer E. Designing attractive workplace health promotion programs. 2021; Available from: https://www.scopus.com/inward/record.uri?eid=2-s2.0-85110885525&doi=10.1108%2fER-10-2020-0451&partnerID=40&md5=38a289043bed154c892b7bf3aa81bc5b

Reason for exclusion: Non adaptation to the objective of the study from the perspective of its scope

Piwowar-Sulej K, Bak-Grabowska D. Non-Permanent employment and employees’ health in the context of sustainable HRM with a focus on Poland. 2020;9(9). Available from: https://www.scopus.com/inward/record.uri?eid=2-s2.0-85089286545&doi=10.3390%2fSOCSCI9070117&partnerID=40&md5=3d77442eafbe633f927f447dafab20c8

Reason for exclusion: Non adaptation to the objective of the study from the perspective of its scope

Ruiz-Dominguez F, Stegeman I, Dolz-López J, Papartyte L, Fernández-Pérez D. Transfer and Implementation Process of a Good Practice in Workplace Health Promotion. International Journal of Environmental Research and Public Health [Internet]. 2021;18(10). Available from: https://www.proquest.com/scholarly-journals/transfer-implementation-process-good-practice/docview/2532494612/se-2

Reason for exclusion: Non adaptation to the objective of the study from the perspective of its scope

Rupp DE, Mallory DB. Corporate Social Responsibility: Psychological, Person-Centric, and Progressing. 2015;2:236. Available from: https://www.scopus.com/inward/record.uri?eid=2-s2.0-84971004601&doi=10.1146%2fannurev-orgpsych-032414-111505&partnerID=40&md5=c5c066aa2822e899a383db0a7d4538dc

Reason for exclusion: Non adaptation to the objective of the study from the perspective of its scope

Scarduzio JA, Geist-Martin P. Workplace wellness campaigns: The four dimensions of a whole-person approach. 2016;186. Available from: https://www.scopus.com/inward/record.uri?eid=2-s2.0-84960337970&doi=10.4324%2f9781315723020&partnerID=40&md5=30a6f9e25354a9e1d7383139d918a69c

Reason for exclusion: Non adaptation to the objective of the study from the perspective of its scope

Tung C-Y, Yin Y-W, Liu C-Y, Chang C-C, Zhou Y-P. Employer and Promoter Perspectives on the Quality of Health Promotion Within the Healthy Workplace Accreditation. 2017;59(7):648. Available from: https://www.scopus.com/inward/record.uri?eid=2-s2.0-85021817476&doi=10.1097%2fJOM.0000000000001057&partnerID=40&md5=5bffbf57511c465b2c182b0e3a302f56

Reason for exclusion: Non adaptation to the objective of the study from the perspective of its scope

Tung C-Y, Yin Y-W, Zhou Y-P, Chang C-C, Lin P-Y, Liu C-Y. An analysis of healthy workplace accreditation and health promotion efforts based on employees’ perspectives. 2018;73(5):329. Available from: https://www.scopus.com/inward/record.uri?eid=2-s2.0-85061580165&doi=10.1080%2f19338244.2017.1365683&partnerID=40&md5=cd7fe9fe91fd9aa1170def264c70f35b

Reason for exclusion: Non adaptation to the objective of the study from the perspective of its scope

Tziraki-Segal C, De Luca V, Santana S, Romano R, Tramontano G, Scattola P, et al. Creating a Culture of Health in Planning and Implementing Innovative Strategies Addressing Non-communicable Chronic Diseases. 2019;4. Available from: https://www.scopus.com/inward/record.uri?eid=2-s2.0-85092273980&doi=10.3389%2ffsoc.2019.00009&partnerID=40&md5=37af1c397457360c3ccd8201cb70dfe4

Reason for exclusion: Non adaptation to the objective of the study from the perspective of its scope

Verbeek J, Pulliainen M, Kankaanpää E. A systematic review of occupational safety and health business cases. 2009;35(6):412. Available from: https://www.scopus.com/inward/record.uri?eid=2-s2.0-73449116112&doi=10.5271%2fsjweh.1355&partnerID=40&md5=a10ec543a8d81d9da91a964e006c1547

Reason for exclusion: The research design is not clearly specified and appropriate for the aims and objectives of the research

Vuontisjärvi T. Corporate social reporting in the European context and human resource disclosures: An analysis of finnish companies. 2006;69(4):354. Available from: https://www.scopus.com/inward/record.uri?eid=2-s2.0-33751117487&doi=10.1007%2fs10551-006-9094-5&partnerID=40&md5=95386c1fa40970c920d29cd53a4eb680

Reason for exclusion: The research design is not clearly specified and appropriate for the aims and objectives of the research

Wofford D, MacDonald S, Rodehau C. A call to action on women’s health: Putting corporate CSR standards for workplace health on the global health agenda. 2016;12(1). Available from: https://www.scopus.com/inward/record.uri?eid=2-s2.0-84997496953&doi=10.1186%2fs12992-016-0206-4&partnerID=40&md5=907c942285f425c3f1b4b43433371396

Reason for exclusion: Lack of originality

Wong K, Chan AHS, Teh P-L. How is work–life balance arrangement associated with organisational performance? A meta-analysis. 2020;17(12):19. Available from: https://www.scopus.com/inward/record.uri?eid=2-s2.0-85086884493&doi=10.3390%2fijerph17124446&partnerID=40&md5=48b4ce7dd76d6ab849c7ba24d1c57334

Reason for exclusion: Non adaptation to the objective of the study from the perspective of its scope

Ybema JF, Evers MS, Van Scheppingen AR. A longitudinal study on the effects of health policy in organizations on job satisfaction, burnout, and sickness absence. 2011;53(11):1257. Available from: https://www.scopus.com/inward/record.uri?eid=2-s2.0-81155139117&doi=10.1097%2fJOM.0b013e318234e2b0&partnerID=40&md5=4350ebf716aa60a1569f6c4a8bbb170c

Reason for exclusion: Non adaptation to the objective of the study from the perspective of its scope

Zhang J. The effect of corporate social responsibility on hotel employee safety behavior during COVID-19: The moderation of belief restoration and negative emotions. Journal of Hospitality and Tourism Management. 2021;46:233–43.

Reason for exclusion: Non adaptation to the objective of the study from the perspective of its scope

Zink KJ, Thul MJ. Corporate health management: Designing and evaluating health in organizations. 2006; Available from: https://www.scopus.com/inward/record.uri?eid=2-s2.0-85083945999&partnerID=40&md5=d6a0a75b23a5fc466753d806cc3827ca

Reason for exclusion: Non adaptation to the objective of the study from the perspective of its scope
